# Efficient delivery of C/EBP beta gene into human mesenchymal stem cells via polyethylenimine-coated gold nanoparticles enhances adipogenic differentiation

**DOI:** 10.1038/srep33784

**Published:** 2016-09-28

**Authors:** Das Joydeep, Yun-Jung Choi, Hideyo Yasuda, Jae Woong Han, Chankyu Park, Hyuk Song, Hojae Bae, Jin-Hoi Kim

**Affiliations:** 1 Dept. of Stem Cell and Regenerative Biology, Humanized Pig Research Center (SRC), Konkuk University, Seoul 143-701, South Korea; 2 Dept. of Bioindustrial Technologies, College of Animal Bioscience and Technology, Konkuk University, Seoul 143-701, South Korea

## Abstract

The controlled differentiation of stem cells via the delivery of specific genes encoding appropriate differentiation factors may provide useful models for regenerative medicine and aid in developing therapies for human patients. However, the majority of non-viral vectors are not efficient enough to manipulate difficult-to-transfect adult human stem cells *in vitro*. Herein, we report the first use of 25 kDa branched polyethylenimine-entrapped gold nanoparticles (AuPEINPs) and covalently bound polyethylenimine-gold nanoparticles (AuMUAPEINPs) as carriers for efficient gene delivery into human mesenchymal stem cells (hMSCs). We determined a functional application of these nanoparticles by transfecting hMSCs with the C/EBP beta gene, fused to EGFP, to induce adipogenic differentiation. Transfection efficacy with AuPEINPs and AuMUAPEINPs was 52.3% and 40.7%, respectively, which was 2.48 and 1.93 times higher than that by using Lipofectamine 2000. Luciferase assay results also demonstrated improved gene transfection efficiency of AuPEINPs/AuMUAPEINPs over Lipofectamine 2000 and polyethylenimine. Overexpression of exogenous C/EBP beta significantly enhanced adipogenesis in hMSCs as indicated by both of Oil Red O staining and mRNA expression analyses. Nanoparticle/DNA complexes exhibited favorable cytocompatibility in hMSCs. Taken together, AuPEINPs and AuMUAPEINPs potentially represent safe and highly efficient vehicles for gene delivery to control hMSC differentiation and for therapeutic gene delivery applications.

Stem cell therapies offer the promise of treating various human diseases, including neurodegenerative disorders, cardiovascular diseases, cancers, and ischemic brain injuries^1^,^2^. Human mesenchymal stem cells (hMSCs) have immunosuppressive properties and can be differentiated into various cell types, including adipocytes, myocytes, chrondocytes, and osteoblasts, because of their multipotent nature^3^,^4^,^5^,^6^,^7^. In addition, the robust expansion capabilities of hMSCs are advantageous for the regeneration of damaged tissues^8^,^9^. Recently, hMSC-based therapies have been extensively applied to adipogenic/soft tissue regeneration^10^,^11^. Prior to transplantation *in vivo*, MSC fate may be better controlled by the lineage-specific differentiation of stem cells *in vitro* via the delivery of specific genes encoding appropriate differentiation factors^12^,^13^,^14^,^15^. However, a safe and efficient gene delivery system for MSCs is currently unavailable. Viral vectors can be used to deliver genes efficiently but their use in clinical applications is limited due to safety concerns and intrinsic limitations of DNA packaging^16^,^17^. Electroporation techniques can also be used to deliver genes efficiently but result in extensive cell death^18^,^19^.

Therefore, non-viral vectors, including inorganic nanoparticles, are emerging as attractive gene delivery vectors, because they are generally biocompatible, easily functionalized, structurally varied, and able to carry miscellaneous genetic materials^20^. Inorganic nanoparticles used for gene delivery include superparamagnetic iron oxide^21^,^22^,^23^, silica nanoparticles^13^,^24^, quantum dots^25^, gold nanoparticles^26^,^27^,^28^,^29^, carbon nanotubes^30^, and calcium phosphate nanoparticles^31^. However, the majority of non-viral vectors are not efficient enough to deliver genes to difficult-to-transfect hMSCs. Over the past decade, gold nanoparticles (AuNPs) have been used in diverse applications in biology and medicine because of their facile synthesis, biocompatibility, tunable size and shape, easy surface modification and bioconjugation, and tunable electronic and optical properties^32^,^33^,^34^,^35^,^36^. However, the efficiency of gene delivery by AuNPs into hMSCs has been poor. In the present study, we modified the surface of AuNPs with 25 kDa branched polyethylenimine (PEI), a commercially available cationic polymer, to enhance their transfection efficiency in difficult-to-transfect cells, such as hMSCs. PEI is a well-studied cationic polymer, which on its own has been used as a non-viral gene delivery vector or more often to modify the surfaces of nanovectors due to their controllable synthesis, highly abundant surface amino groups, and their ability to compact large amounts of nucleic acids^13^,^30^,^37^,^38^,^39^,^40^,^41^.

Herein, we demonstrate that by combining the advantages of PEI as a gene delivery vector and the remarkable roles played by AuNPs in gene delivery applications, we can prepare a single-particle system (AuNPs/PEI conjugates) with high gene delivery efficiency. The transfection efficiency of polyethylenimine-entrapped gold nanoparticles (AuPEINPs) and covalently bound polyethylenimine-gold nanoparticles (AuMUAPEINPs) were compared to the transfection efficiency by using commercially available transfection reagents in hMSCs. In addition, the potential therapeutic applications of AuPEINPs and AuMUAPEINPs as a gene delivery vector were evaluated by determining their ability to transfect hMSCs with the human CCAAT/enhancer binding protein beta (C/EBPβ) gene, which encodes a key transcriptional regulator of adipogenic differentiation^42^,^43^,^44^,^45^ fused to enhanced green fluorescent protein (EGFP-C/EBPβ), and induce the differentiation of hMSCs into adipocytes.

## Results and Discussion

### Construction of pEGFP-C/EBPβ vector and characterization of AuPEINPs, and AuMUAPEINPs

In the present study, we prepared an expression vector encoding pEGFP-C/EBPβ, whose expression was driven by a CMV promoter (Supplementary Figure 1), to determine transgene expression efficiency and to induce adipogenic differentiation of hMSCs. C/EBPβ is a key transcriptional regulator of adipogenic differentiation^42^,^43^,^44^,^45^. To test the efficient delivery of pEGFP-C/EBPβ into hMSCs, we synthesized two types of gold nanoparticles: (i) polyethylenimine-entrapped gold nanoparticles (AuPEINPs) and (ii) covalently bound polyethylenimine-gold nanoparticles (AuMUAPEINPs). AuPEINPs were synthesized by the sodium borohydride reduction method in the presence of polyethylenimine (PEI). AuMUAPEINPs were prepared with the use of 11-mercaptoundecanoic acid as a spacer, which can bind gold nanoparticles through thiol groups and make an amide bond with PEI. The formation of 1-[(11-sulfanylundecanoyl)oxy] pyrrolidine-2,5-dione was confirmed by mass spectral analysis (Supplementary Figure 2); we observed a molecular ion peak at m/z of 315 and another peak at m/z of 201 due to ester bond breaking. PEI was then added to the activated acid to form 11-mercaptoundecanoic acid-polyethylenimine conjugates (MUAPEI). MUAPEIs were further resuspended in water and used to coat gold nanoparticles generated *in situ* via the reduction of HAuCl_4_·3H_2_O using NaBH_4_. AuPEINPs and AuMUAPEINPs were purified by centrifugal filtration using 50 kDa MW cutoff membrane filters (repeated twice) to remove excess PEI and MUAPEI, respectively.

**Figure 1:**
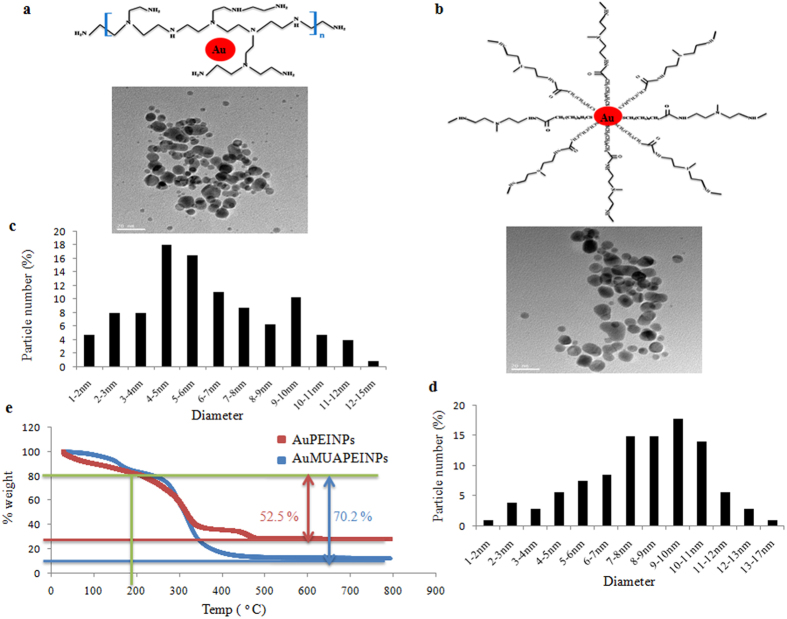
Characterization of nanoparticles. (**a**) Schematic presentation and TEM image of AuPEINPs. (**c**) Particle diameter distribution of AuPEINPs. (**b**) Schematic presentation and TEM image of AuMUAPEINPs. (**d**) Particle diameter distribution of AuMUAPEINPs. (**e**) Weight loss curves (TGA) of AuPEINPs and AuMUAPEINPs.

Synthesized AuPEINPs and AuMUAPEINPs were characterized by transmission electron microscopy (TEM), UV-VIS spectroscopy and dynamic light scattering (DLS) analysis. TEM analysis revealed nearly spherical particles of synthesized AuPEINPs and AuMUAPEINPs with primary core diameters in the range of 4–6 nm and 7–11 nm, respectively (Fig. 1a–d). Both these PEI-conjugates exhibited the plasmon band of gold nanoparticles with λ_max_ ~ 500 nm, similar as reported by Thomas and Klibanov^46^. Distinct absorption bands at 525 and 535 nm were observed by UV spectroscopy of synthesized AuPEINPs and AuMUAPEINPs, respectively, which indicated a larger particle size for the latter sample but was otherwise devoid of impurity peaks (Supplementary Figure 3). In addition, we determined the hydrodynamic diameter of synthesized AuPEINPs and AuMUAPEINPs using dynamic light scattering (DLS) and calculated surface zeta potentials. The average diameter of AuPEINPs and AuMUAPEINPs were 48.55 ± 0.96, and 60.06 ± 0.67 nm, respectively (Supplementary Figure 4), as determined by DLS. Nanoparticles appear larger by DLS compared to by TEM analysis because of the solvation/hydration of nanoparticles. Zeta potential measurements indicated high positive surface charge of synthesized AuPEINPs (32.8 ± 1.9) and AuMUAPEINPs (40.9 ± 0.9) (Supplementary Figure 4). The concentration and the number of nanoparticles in AuPEINPs and AuMUAPEINPs stock solutions were calculated following the method of Handel *et al*.^47^ and Liu *et al*.^48^, respectively.

The surface modification of gold nanoparticles was also checked by elemental analysis. A high percentage of elemental carbon and nitrogen indicated the presence of PEI in AuPEINPs (Supplementary Table 1). Similarly, the presence of carbon, nitrogen and sulfur also indicated the presence of MUAPEI in AuMUAPEINPs (Supplementary Table 1). Further, we have performed thermogravimetric analysis (TGA) to measure the grafting density of the surface coating agents (PEI and MUAPEI) on gold nanoparticles surface. The weight loss graphs show few-step profiles for AuPEINPs and AuMUAPEINPs (Fig. 1e). A pure MUAPEI sample, analyzed for comparison, displayed a major derivative peak around 350 °C and around 75% mass loss between 200 and 800 °C (Supplementary Figure 5). On the other hand, Buchman *et al*.^49^, reported that pure 25 kd branched PEI displayed also a derivative peak around 350 °C and around 86% mass loss between 200 and 800 °C. However, the first 27−200 °C nanoparticle weight losses corresponded to the evaporation of water and adsorbed low weight solvents or reagents^49^. Additional observed weight loss steps between 200 and 800 °C indicates the actual organic phase materials attachment on nanoparticle surface. Therefore, we have only considered the weight loss between 200 and 800 °C for grafting density calculation. For AuPEINPs, around 52.5% of PEI was attached on nanoparticle surface with grafting density of 0.42 molecules/nm^2^, where as, for AuMUAPEI, around 70.2% MUAPEI was attached on nanoparticle surface with grafting density of 1.62 molecules/nm^2^ (Fig. 1e, Supplementary Table 2). However, the number of MUAPEI molecules (412) per nanoparticle for approximately 9 nm diameter Au nanoparticles was found to be higher compared with the number of PEI molecules (34) per nanoparticle for approximately 5 nm diameter Au nanoparticles, although the molecular weight of the ligands was almost similar (Supplementary Table 2). A similar size-dependent observation was made by Rahme *et al*.^50^, using mPEG10000-SH ligands to quantify the number of ligands grafted onto 15 to 115 nm diameter Au nanoparticles. The fact that MUAPEI display a higher density of ligand attachment has been attributed to their strong covalent-like bonding with gold due to the presence of thiol groups as well as large surface area of gold nanoparticles (mean diameter 9 nm) available for ligand attachment. On the other hand, PEI can be conjugated with gold surface via weak non-covalent interaction by the use of electron pair on nitrogen as well as smaller surface area of gold nanoparticles (mean diameter 5 nm) available for ligand attachment.

### Characterization of the nanoparticle-plasmid DNA complexes

The optical absorbance, hydrodynamic diameter and surface zeta potentials of nanoparticle-pDNA complexes were also determined. Broad bands spanning 520–535 nm and 520–540 nm were observed for AuPEINPs-pEGFP-C/EBPβ and AuMUAPEINPs-pEGFP-C/EBPβ complexes (Supplementary Figure 3), indicating even larger particle sizes than of the corresponding nanoparticles alone. DLS analysis confirmed increases in particle sizes; the average diameters of AuPEINPs and AuMUAPEINPs increased by approximately 23 nm and 30 nm, respectively, when complexed with pDNA (Supplementary Table 3). The zeta potentials for AuPEINPs-pEGFP-C/EBPβ (+28.6 ± 2.7 mV) and AuMUAPEINPs-pEGFP-C/EBPβ (+21.7 ± 0.6 mV) complexes decreased slightly due to the association with negatively charged DNA (Supplementary Table 3). Large positive zeta potentials and hydrodynamic sizes of nanoparticle-pDNA complexes <100 nm are ideal for their successful cellular internalization and subsequent gene delivery.

### pDNA binding affinity of AuPEINPs and AuMUAPEINPs

**Figure 2:**
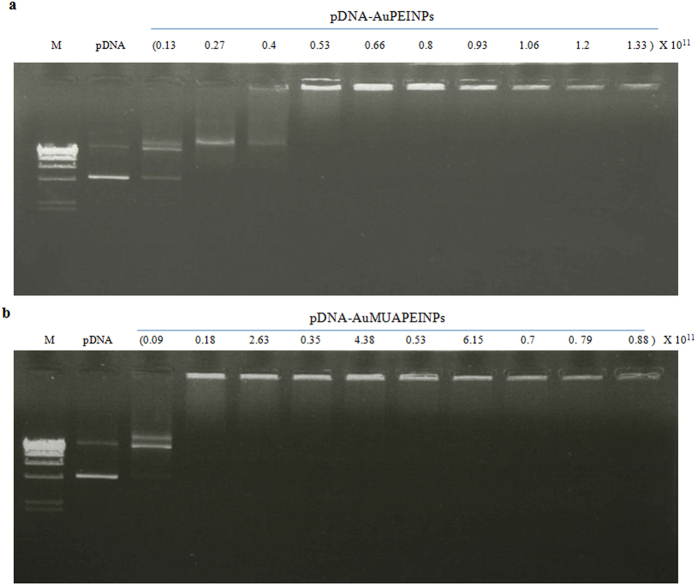
Agarose gel retardation assay. (**a**) AuPEINPs-pDNA complexes were prepared at different ratios of particle numbers to 1 μg of pDNA. (**b**) AuMUAPEINPs-pDNA complexes were prepared at different ratios of particle numbers to 1 μg of pDNA. M: DNA marker. The number of nanoparticles used is shown on the top of each lane.

One of the most important factors that determine the efficiency of gene delivery is the association of the transfection agent with pDNA. Cationic polymer vectors compact pDNA via electrostatic interactions^39^,^40^,^51^. Herein, to assess nucleic acid binding ability, AuPEINPs and AuMUAPEINPs were mixed with pEGFP-C/EBPβ (1 μg) at varying concentration of AuPEINPs and AuMUAPEINPs, and analyzed by agarose gel (1.0% w/v) retardation assays. AuPEINPs and AuMUAPEINPs retarded 1 μg of pDNA at particle numbers 0.53 × 10^11^ and 0.18 × 10^11^, respectively or above, indicating successful DNA binding via electrostatic interactions (Fig. 2).

### Gene transfection efficiency

**Figure 3:**
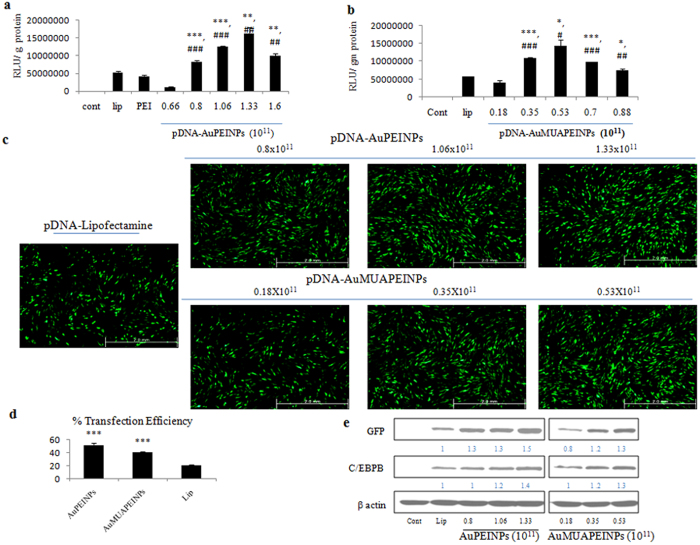
Gene transfection efficiency measurements. (**a**,**b**) Transfection efficiency of AuPEINPs and AuMUAPEINPs measured by luciferase reporter assays. All values are expressed as mean ± SD. (**c**) Fluorescence micrographs of cells transfected with pEGFP-C/EBPβ. (**d**) Quantification of GFP-positive cells by flow cytometry analysis. Values are expressed as mean ± SD. (**e**) Western blot analysis of GFP and C/EBPB proteins 48 h after transfection with pEGFP-C/EBPβ. Densitometric analyses were performed using Image J software, and intensity values were normalized with respect to β-actin. 1 μg of pDNA was used for all transfection experiments. *p < 0.05, **p < 0.01, and ***p < 0.001 versus Lipofectamine 2000 transfected group. ^#^p < 0.05, ^##^p < 0.01, and ^###^p < 0.001 versus PEI transfected group.

Without impacting their viability, stem cells are difficult to transfect with non-viral vectors, including with lipid and polymeric agents. The transfection efficiency of Lipofectamine 2000, PEI, AuPEINPs, and AuMUAPEINPs were determined using luciferase reporter assays, which is a sensitive method for assessing transgene delivery and expression^52^. The transfection efficiency of 1 μg of pGL3-Control (after 48 h) was the highest with AuPEINPs and AuMUAPEINPs at particle numbers of 1.33 × 10^11^ and 0.53 × 10^11^, respectively (Fig. 3a,b). At nontoxic doses, the transfection efficiency with AuPEINPs was 3 and 4 times higher than with Lipofectamine 2000 and unmodified PEI, respectively (Fig. 3a). Similarly, the transfection efficiency with AuMUAPEINPs was 2.5 and 3.3 times higher than with Lipofectamine 2000 and unmodified PEI, respectively (Fig. 3b). Thomas and Klibanov^46^ also reported that PEI-conjugated gold nanoparticles exhibited higher transfection efficiency compared with free PEI in COS-7 cells. These results demonstrate the effectiveness of AuPEINPs and AuMUAPEINPs over the commercial transfection agents as gene delivery vectors for difficult-to-transfect hMSCs.

However, from Fig. 2, we have observed that AuPEINPs and AuMUAPEINPs retarded 1 μg of pDNA at particle numbers 0.53 × 10^11^ and 0.18 × 10^11^, respectively or above. Thus, for AuPEINPs and AuMUAPEINPs, the fraction of nanoparticles just sufficient to bind 1 μg of the plasmids is approximately (0.53 × 10^11^/1.33 × 10^11^ × 100) 40% and (0.18 × 10^11^/0.53 × 10^11^ × 100) 34% respectively. It has been proposed that endosomal and cytoplasmic degradation of the plasmid DNA is a key barrier to transfection following endocytosis^53^. Therefore, we can assume that the remaining ((1.33–0.53) × 10^11^/1.33 × 10^11^ × 100) 60% (AuPEINPs) and ((0.53–0.18) × 10^11^/0.53 × 10^11^ × 100) 66% (AuMUAPEINPs) of the used nanoparticles are essential to provide more positive surface charge which is necessary for better compaction of plasmid DNA inside the complexes, and subsequent stability of the plasmid DNA from enzymatic degradation against nucleases inside the cells.

### Transfection with EGFP-C/EBPβ gene

For subsequent transfections, the optimal range of particle numbers for AuPEINPs and AuMUAPEINPs were chosen based on the performance of these nanoparticles in luciferase reporter assays. In addition, we tested only Lipofectamine 2000 in subsequent transfections with pEGFP-C/EBPβ because hMSCs are more efficiently transfected with Lipofectamine 2000 than with PEI, which has also been observed in previous studies^51^,^54^. Cells were incubated with nanoparticle-pEGFP-C/EBPβ complexes and gene expression was analyzed by fluorescence microscopy, flow cytometry and western blotting 48 h after transfection. Together, these analyses demonstrated that hMSCs are more efficiently transfected with AuPEINPs and AuMUAPEINPs than with Lipofectamine 2000 (Fig. 3c–e). The transfection efficiencies with AuPEINPs and AuMUAPEINPs were 52.3% and 40.7%, respectively, which are 2.48 and 1.93 times higher than with Lipofectamine 2000 (Fig. 3d). Protein expression levels of C/EBPβ also indicated that transfections with AuPEINPs and AuMUAPEINPs were more efficient than with Lipofectamine 2000 (Fig. 3e). The higher transfection efficiency of AuPEINPs compared to AuMUAPEINPs is attributed due to its smaller size and increased positive charge density upon complex formation with pDNA.

Tierney *et al*.^55^,^56^ and several other researchers^57^,^58^,^59^ investigated the transfection efficiency of unmodified polyethyleneimine (PEI) in rat, human and porcine mesenchymal stem cells (MSCs). In their reports they showed that PEI could transfect around 45%, 25% and 20% MSCs derived from rats, human and porcine origin respectively^55^,^56^,^57^,^58^,^59^. Therefore, it is obvious that the transfection efficiency of PEI in MSCs depends on the origin of the species. In our current study, we used the human derived MSCs and the transfection efficiency of AuPEINPs and AuMUAPEINPs were 52.3% and 40.7%, respectively, which are much higher than the transfection efficiency of unmodified PEI (25%) in human derived MSCs^56^. These results demonstrate that AuPEINPs and AuMUAPEINPs might be used for the successful delivery of a wide range of genes in difficult-to-transfect hMSCs for potential therapeutic applications.

### Adipogenic differentiation following pEGFP-C/EBPβ transfection

**Figure 4:**
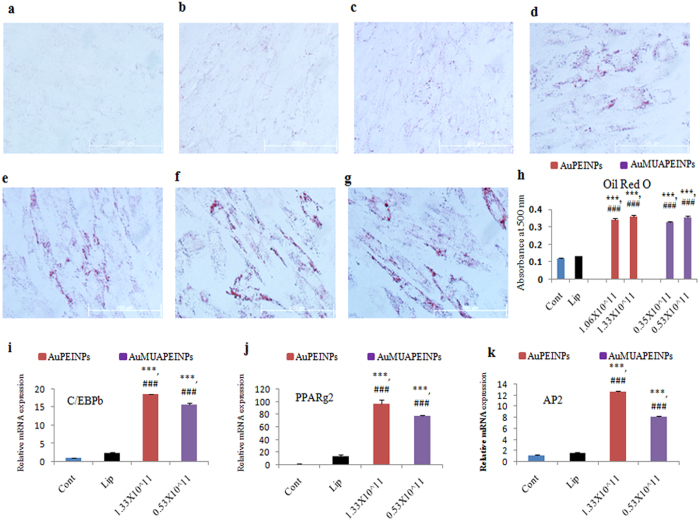
Oil Red O staining and gene expression of adipogenic differentiation markers 7 days after transfecting the C/EBPβ gene. (**a**) Oil Red O staining of non-treated control cells cultured in growth media. (**b**) Oil Red O staining of non-treated control cells cultured in ADM. (**c**) Oil Red O staining of cells expressing the C/EBPβ gene transfected with Lipofectamine 2000 and cultured in ADM. (**d**) and (**e**) Oil Red O staining of cells expressing the C/EBPβ gene transfected with AuPEINPs (1.06 × 10^11^ and 1.33 × 10^11^ number of particles, respectively) and cultured in ADM. (**f**) and (**g**) Oil Red O staining of cells expressing the C/EBPβ gene transfected with AuMUAPEINPs (0.35 × 10^11^ and 0.53 × 10^11^ number of particles, respectively) and cultured in ADM. (**h**) Quantitative analysis of lipid droplet formation. (**i**–**k**) qRT-PCR analysis of the relative mRNA expressions of C/EBPβ, PPARγ2, and AP2 in cells transfected with the C/EBPβ gene and cultured in ADM. All values are expressed as mean ± SD. *p < 0.05, **p < 0.01, and ***p < 0.001 versus the control non-transfected group. ^#^p < 0.05, ^##^p < 0.01, and ^###^p < 0.001 versus Lipofectamine transfected group.

To demonstrate the potential application of nanoparticle-mediated gene transfections of hMSCs, hMSC cells were transfected with a pDNA encoding the EGFP-C/EBPβ gene, a key transcriptional regulator of adipogenic differentiation^42^,^43^,^44^,^45^. Induction of hMSC adipogenic differentiation by the transfection of C/EBPβ genes has been reported previously^39^. To confirm C/EBPβ-induced stem cell adipogenic differentiation, Oil Red O staining was performed to stain lipid droplets inside differentiated hMSCs. After transfection, hMSCs were cultured in adipogenic differentiation medium (ADM) for either 7 or 14 days. Non-transfected (control) cultures were maintained either in growth media or in ADM for either 7 or 14 days. After 7 days of culture in ADM, lipid droplet formation was 3 times greater in hMSC cells transfected with AuPEINPs and AuMUAPEINPs vectors than in hMSC cells transfected with Lipofectamine 2000 or control cells (Fig. 4a–h). Furthermore, we measured C/EBPβ mRNA expression, and the expression of other adipogenic differentiation-specific genes, such as peroxisomal proliferator-activated receptor γ2 (PPARγ2) and adipocyte protein 2 (AP2), 7 days after transfecting the C/EBPβ gene. PPARγ2 is a key adipogenic transcription factor, whose expression is directly induced by C/EBPβ^60^. In addition, PPARγ2 activates a large number of genes related to fatty acid metabolism and adipogenesis, including AP2, which is expressed in matured adipocytes^61^. After 7 days of culture in ADM, C/EBPβ gene expression was higher in hMSCs transfected with AuPEINPs and AuMUAPEINPs vectors than in cells transfected with Lipofectamine 2000 or in control cells (Fig. 4i). The expression of PPARγ2 and AP2 were also higher in cells transfected with AuPEINPs and AuMUAPEINPs vectors than in cells transfected with Lipofectamine 2000 or control cells (Fig. 4j,k). Therefore, transfection with PEI-modified gold nanoparticles not only showed enhanced transfection efficiency in difficult to transfect hMSCs over the commercially available transfection reagents but also retained the function of the transfected gene and maintained its higher gene level for long time. After 14 days of culture in ADM, lipid droplet formation was still 24–28% and 16–20% higher in transfected hMSCs than in control non-transfected and Lipofectamine 2000 transfected cells, respectively, as measured by quantitative Oil Red O staining (Supplementary Figure 6). These results demonstrate that PEI-modified gold nanoparticles can be used as robust gene delivery vectors to enhance traditional differentiation-medium-based approaches to modulate the lineage-specific differentiation of hMSCs.

### Stability of nanoparticle-plasmid DNA complexes

The stability of AuPEINPs-pDNA and AuMUAPEINPs- pDNA complexes over the period of incubation was checked as described by Lee *et al*.^62^. Cells were treated with the nanoparticle-pDNA complexes for 6 hrs in presence of media and nanoparticle aggregation in the media was checked by optical microscopic analysis. For AuPEINPs, the nanoparticles-pDNA complexes were stable without aggregation upto 2.65 × 10^11^ nanoparticle concentration, which is double of optimum concentration showing maximum transfection efficiency (Supplementary Figure 7). Similarly, for AuMUAPEINPs, the nanoparticle-pDNA complexes were stable upto 0.88 × 10^11^ nanoparticle concentrations (Supplementary Figure 8). However, nanoparticle aggregation was found at 1.05 × 10^11^ nanoparticle concentration, which is double of optimum concentration showing maximum transfection efficiency (Supplementary Figure 8). Therefore, we can conclude that both kinds of PEI-modified gold nanoparticles are stable at least upto their respective optimum concentrations showing maximum transfection efficiency.

### Cellular uptake of nanoparticle-plasmid DNA complexes

**Figure 5:**
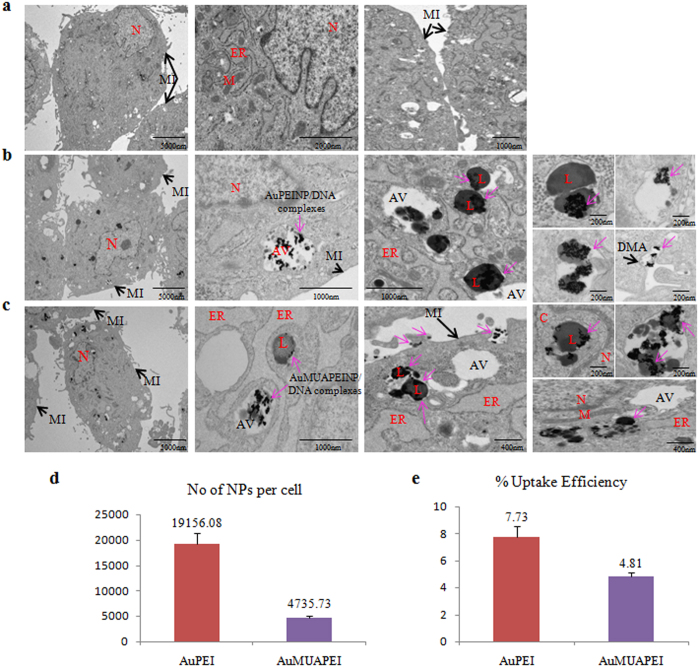
Cellular localization and quantitative determination of cellular uptake of nanoparticle-DNA complexes. (**a**) TEM analysis of control non-treated cells. (**b**,**c**) TEM analysis for cellular localization of AuPEINPs-pDNA and AuMUAPEINPs-pDNA complexes. M: mitochondria; N: nuclei; C: cytoplasm; AV: autophagic vesicles; DMA: double-membrane autophagosomes; L: lysosomes; MI: membrane integrity; ER: endoplasmic reticulum; pink arrow: nanoparticle/DNA complexes. (**d**) The number of nanoparticles internalized per cell determined by ICP-MS. (**e**) % uptake efficiency of nanoparticles into cells. All values are expressed as mean ± SD.

In general, the cellular uptake pathways of allogeneic materials, which include phagocytosis, endocytosis (clathrin-dependent, caveolae-dependent, or clathrin/caveolae-independent endocytosis), and macropinocytosis, depend on the cells and transfection agent used^63^,^64^. To investigate the cellular uptake of the nanoparticles-pDNA complexes, we tracked the intracellular distribution of the nano-complexes within the cells by TEM analysis following transfection of the cells with AuPEINPs-pDNA and AuMUAPEINPs-pDNA for 12 hrs. The nanoparticles-pDNA complexes were internalized and clustered in the cytoplasm (Fig. 5a–c). The endocytosed AuPEINPs-pDNA and AuMUAPEINPs-pDNA complexes in the hMSCs were present in lysosomes and autophagic vesicles, but did not reach the nucleus (Fig. 5a–c). Endocytosis of PEI-conjugated gold nanoparticles-pDNA complexes in COS-7 cells were also reported by Thomas and Klibanov^46^. Endo-lysosomal pathways are the most common intracellular mechanism of nanoparticle sequestration and degradation following endocytosis of nanoparticles^65^. In addition, gold and several other non-degradable nanoparticles can also induce autophagy^66^,^67^ which involves intercellular degradation of foreign invaders through the lysosomal machinery in which cytoplasmic materials are degraded by being sequestered into double membrane vesicles and transported to lysosomes^68^,^69^. Treatment with both kinds of nanoparticles-pDNA complexes neither affects cell membrane integrity nor induces structural damage of the intracellular organelles. However, the endoplasmic reticulum of the AuMUAPEINPs-pDNA treated hMSCs were swollen compared with AuPEINPs-pDNA treated and control groups.

We have also checked the cellular membrane integrity by Lactate dehydrogenase (LDH) release in to cell culture media following treatment with nanoparticles-pDNA complexes for 12 hrs. Treatment with nanoparticles-pDNA complexes did not cause cell membrane damage as there was no LDH release in to the culture medium compared with control group (Supplementary Figure 9). Several other researchers have also shown that PEI-modified metal nanoparticles, such as, gold, iron oxide and quantum dots entered into cells via endocytosis^25^,^51^,^70^. Therefore, we can assume that the positively charged nanoparticle-pDNA complexes bind effectively with negatively charged cell membrane, thereby enhancing cellular uptake via endocytosis without rupturing cell membrane^51^.

Further, we have calculated the amount of gold nanoparticles entered into the cells by ICP-MS analysis following treatment of the hMSCs with AuPEINPs (1.33 × 10^11^) and AuMUAPEINPs (0.53 × 10^11^) complexed with 1 μg pDNA for 6 hrs. For AuPEINPs, approximately 19156 nanoparticles entered per cell, whereas for AuMUAPEINPs, approximately 4736 nanoparticles entered per cell (Fig. 5d). As expected, AuPEINPs entered into the cells with a higher efficiency (7.73%) than that of AuMUAPEINPs (4.81%) because of the smaller size of the former (Fig. 5e). All these results demonstrate the efficient delivery of the nanoparticles-pDNA complexes into hMSCs.

### Mechanism of cellular uptake pathways

**Figure 6:**
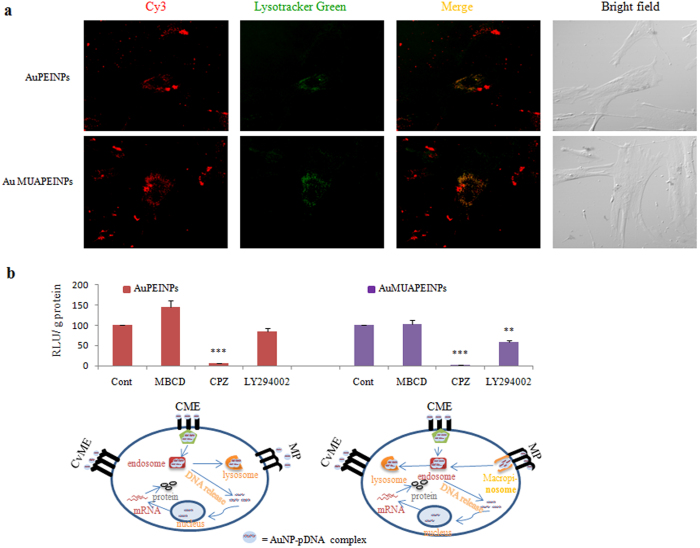
Intracellular distribution and cellular uptake pathways of nanoparticle-DNA complexes. (**a**) Confocal micrographs of the intracellular distribution and localization of gold nanoparticle-Cy3-DNA complexes 12 h after transfection. Green regions indicate lysosomes and endosomes stained with LysoTracker Green. Red regions indicate Cy3-DNA. Yellow regions indicate co-localization of nanoparticle-Cy3-DNA complexes with endosomes/lysosomes. (**b**) The mechanism of cellular uptake of gold nanoparticle-pDNA complexes by hMSCs was investigated by pretreating cells with several endocytosis inhibitors prior to transfecting with the Luc gene. Cells were either cultured at 37 °C as a negative control (Cont) or pretreated with MBCD, chlorpromazine, or LY294002. All values are expressed as mean ± SD. *p < 0.05, **p < 0.01, and ***p < 0.001 versus the non-treated control group.

To confirm the endocytotic pathways, the transfected cells were imaged with confocal microscopy to determine the intracellular distribution of gold nanoparticle-Cy3-DNA complexes 12 h post-transfection. Acidic organelles of live cells, such as endosomes and lysosomes, were stained with LysoTracker Green. Gold nanoparticle-Cy3-DNA complexes (red) were localized within endosomes/lysosomes (green), indicating that nanovector/DNA complexes were internalized mostly through endocytosis (Fig. 6a). Then protonation of tertiary amino groups of PEI on the gold nanoparticle surface within endosomes induces their release into the cytoplasm due to proton sponge mechanisms^71^.

The mechanism of cellular uptake of gold nanoparticle-pDNA complexes into hMSCs was investigated by pretreating cells with several endocytosis inhibitors prior to transfections in luciferase reporter assays. The cellular uptake of nanoparticle-pDNA complexes can be assessed by the expression of a transgene unless the inhibitors used induce toxicity^72^,^73^,^74^. The inhibitors in our study did not induce significant toxicity; therefore, it is likely that the inhibitors themselves did not directly influence gene expression, and any decrease in transgene expression is likely caused by an effect on cellular uptake of the nanoparticle-pDNA complexes. Cells were pretreated with several endocytosis inhibitors, including 5 μg/mL chlorpromazine (inhibitor of clathrin-mediated endocytosis), 1 mM methyl-β–cyclodextrin (MBCD, suppressor or inhibitor of caveolae-mediated endocytosis), and 65 μM LY294002 (inhibitor of macropinocytosis)^73^,^75^,^76^. After pretreating cells with the different inhibitors, cells were then transfected with AuPEINPs-pGL3 and AuMUAPEINPs-pGL3 complexes (using 1 μg pGL3-Control) at particle numbers of 1.33 × 10^11^ and 0.53 × 10^11^, respectively for 6 h. Pretreatment with MBCD did not reduce luciferase activity in cells transfected with either AuPEINPs or AuMUAPEINPs (Fig. 6b). In contrast, pretreatment with chlorpromazine reduced luciferase activity in cells transfected with AuPEINPs or AuMUAPEINPs by 95%, and 99%, respectively, compared to non-treated cells (Fig. 6b). LY294002 pretreatment reduced luciferase activity in cells transfected with AuPEINPs or AuMUAPEINPs by 15% and 40%, respectively, compared to non-treated cells (Fig. 6b). Nanoparticle size is a crucial parameter in determining cellular uptake pathways^77^,^78^; smaller particles are taken up into cells through both clathrin and caveolae-mediated endocytosis, whereas larger particles are taken up mostly through macropinocytosis^78^,^79^. Here, AuPEINPs-pDNA and AuMUAPEINPs-pDNA complexes were selectively taken up by hMSCs through clathrin-mediated endocytosis. AuMUAPEINPs-pDNA complexes were taken up through macropinocytosis to some extent likely due to their larger size compared to AuPEINPs-pDNA complexes.

### Cytotoxicity measurement

**Figure 7:**
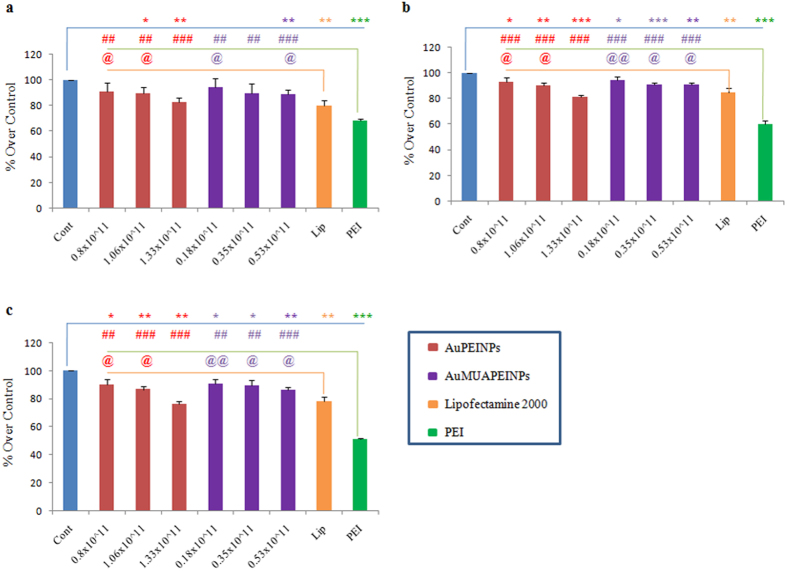
Cell viability assay. Cell viability relative to the untreated control (100%) in hMSCs cells. Cells were treated with different concentrations of AuPEINPs-pEGFP-C/EBPβ and AuMUAPEINPs- pEGFP-C/EBPβ complexes for 6 h and cell viability was measured after 48 h (**a**), 72 h (**b**) and 96 h (**c**) using the Cell Counting Kit-8 (CCK-8). All values are expressed as mean ± SD. *p < 0.05, **p < 0.01, and ***p < 0.001 versus the non-treated control group. ^#^p < 0.05, ^##^p < 0.01, and ^###^p < 0.001 versus the PEI-treated group. ^@^p < 0.05, ^@@^p < 0.01, and ^@@@^p < 0.001 versus the Lipofectamine -treated group.

For gene therapy applications, the cytocompatibility of the gene delivery vector is important to consider. Stem cells are difficult to transfect with non-viral vectors, including lipid and polymeric agents, without affecting their viability^80^,^81^,^82^,^83^,^84^,^85^. Similarly, electroporation can be used to transfect cells with high efficiency but often results in extensive cell death^18^,^19^. Highly efficient and biocompatible non-viral vectors are attractive options for their safety in gene therapy studies. The cytotoxicity of AuPEINPs-pEGFP-C/EBPβ and AuMUAPEINPs-pEGFP-C/EBPβ complexes was evaluated within the concentration range of 0.8 × 10^11^ to 1.33 × 10^11 ^nanoparticles/500 μL and 0.18 × 10^11 ^to 0.53 × 10^11^ nanoparticles/500 μL, respectively, for 1 μg of pDNA, which corresponds to equivalent concentrations for gene transfection experiments using cell viability assay. Besides, we have also compared the cell viability loss following PEI-modified gold nanoparticles-pDNA treatment with Lipofectamine 2000 and unmodified PEI treatment groups. At the highest concentration of AuPEINPs (1.33 × 10^11^ nanoparticles/500 μL), the cell viability was reduced upto approximately 82.5%, 81% and 76% after 48, 72 and 96 hrs respectively (Fig. 7a–c). Similarly, at the highest concentration of AuMUAPEINPs (0.53 × 10^11^ nanoparticles/500 μL), the cell viability was reduced upto approximately 90%, 90% and 87% after 48, 72 and 96 hrs respectively (Fig. 7a–c). However, the cell viability was still significantly higher for both kinds of PEI-modified gold nanoparticles at the highest administered concentration compared with unmodified PEI transfection groups. For the AuPEINPs, there was no significant difference in the cell viability at the highest administered concentration compared with Lipofectamine 2000 transfection groups after 48, 72 and 96 hrs (Fig. 7a–c). On the other hand, For the AuMUAPEINPs, the cell viability was still significantly higher at the highest administered concentration compared with Lipofectamine 2000 transfection groups after 48, 72 and 96 hrs (Fig. 7a–c). Our results clearly demonstrate that both the AuPEINPs and AuMUAPEINPs were less cytotoxic at their respective optimum concentration showing maximum transfection efficiency compared with unmodified PEI. Similarly, AuMUAPEINPs were less cytotoxic at its optimum concentration showing maximum transfection efficiency compared with Lipofectamine 2000, whereas, for AuPEINPs, there was no significant difference in the cytotoxicity at its optimum concentration showing maximum transfection efficiency compared with Lipofectamine 2000. Therefore, the greater biocompatibility of AuPEINPs and AuMUAPEINPs vectors make them ideal for use in gene therapy studies.

## Conclusions

In summary, we prepared polyethylenimine-entrapped gold nanoparticles (AuPEINPs) and covalently bound polyethylenimine-gold nanoparticles (AuMUAPEINPs) for gene delivery into hMSCs. Both AuPEINPs and AuMUAPEINPs were small in size and had high positive zeta potentials. These nanoparticles could effectively compact pDNA to form nanoparticle-pDNA complexes with sizes <100 nm and maintain positive zeta potentials that were suitable for cellular internalization. Two pDNAs, encoding either Luc or EGFP- C/EBPβ, were used in this study to evaluate the transfection efficiency of gold nanoparticles compared to commercial transfection agents. Transfection efficiencies were consistently higher with AuPEINPs and AuMUAPEINPs than with Lipofectamine 2000 and PEI. Higher transfection efficiencies with the C/EBPβ gene resulted in greater differentiation rates of hMSCs into adipocytes. We further investigated the cellular uptake pathways of nanovector-DNA complexes by TEM, ICP-MS analysis, imaging the intracellular distribution of nanovector-Cy3-DNA complexes and by pretreating cells with selective endocytosis inhibitors in luciferase reporter assays. Our results demonstrated that AuPEINPs-DNA complexes were internalized through clathrin-mediated endocytosis, whereas AuMUAPEINPs-DNA complexes were internalized through primarily clathrin-mediated endocytosis and to some extent through macropinocytosis. The higher transfection efficiency of AuPEINPs compared to AuMUAPEINPs is due to its smaller size and increased positive charge density. Based on their favorable cytocompatibility and improved gene transfection efficiency, AuPEINPs and AuMUAPEINPs represent ideal non-viral vectors for difficult-to-transfect cell types in gene therapy applications.

## Material and Methods

### Materials

Gold(III) chloride trihydrate, branched polyethylenimine (25 kDa), sodium borohydrite, 11-mercaptoundecanoic acid, N-hydroxysuccinimide, and fetal bovine serum (FBS) were purchased from Sigma–Aldrich (St. Louis, MO, USA). Penicillin-streptomycin solution, trypsin-EDTA solution, and alpha-MEM, were obtained from Life Technologies (GIBCO, Grand Island, NY, USA). Lipofectamine 2000 and LysoTracker Green DND-L7526 were purchased from Invitrogen (Carlsbad, CA, USA). Plasmid DNA, pGL3-Control, and the Luc assay kit were obtained from Promega Corp (Madison, WI, USA). The bicinchoninic acid (BCA) protein assay system was obtained from Thermo Scientific (Rockford, IL, USA). The antibodies used for immunoblotting were against EGFP (Cell Signaling Technology, Beverly, MA, USA), C/EBPβ (Santa Cruz Biotechnology Inc., Santa Cruz, CA, USA), and beta-actin (Abcam, Cambridge, MA, USA).

### Construction of human pEGFP- C/EBPβ plasmid

The coding region of human C/EBPβ was amplified by PCR using genomic DNA of human colon cancer cells, HCT116 (obtained from Korean Cell Line Bank) as the template, and the primers 5′-ctcgagatatgcaacgcctggtggcctgggac-3′ and 5′-ggatccctagcagtggccggaggaggcgag-3′. The ~1000 bp amplified fragment was cloned into the T Easy vector (Promega, USA) and then subcloned into the *Xho*I/*Bam*HI sites of the pEGFP-C1 vector (Clontech Lab., USA). The sequence was confirmed by dideoxy sequencing method (Macrogen, South Korea).

### Construction of Cyanine 3-labeled DNA

Cyanine 3 (Cy3)-labeled 876 bp DNA fragments (a partial sequence of puromycin-resistant gene) was prepared by polymerase chain reaction (PCR) using 5′Cy3-labeled primers (Macrogen, Korea); GTTTGCGTATTGGGCGCTC, TTAGTCGGGGCTCACTCCTACAG, and pGL4.82 plasmid (Promega, USA) were used as templates. When necessary, highly labeled fragments were obtained by using Cy3-labeled dCTP (PerkinElmer, USA), in place of dCTP, during the PCR reaction. The resultant PCR fragments were purified by 1.0% agarose gel electrophoresis.

### Preparation of polyethylenimine-entrapped gold nanoparticles(AuPEINPs)

Polyethylenimine-entrapped gold nanoparticles (AuPEINPs) were synthesized using the sodium borohydride reduction method in the presence of Polyethylenimine (PEI). Briefly, 25 mL of 1.4 mM HAuCl_4_·3H_2_O and 1 mL of 1% PEI were mixed and stirred vigorously for 30 min at room temperature. After 30 min, 1 mL of 10 mM NaBH_4_ solution was added and stirred overnight. Upon addition of NaBH_4_ solution, the yellow color of the HAuCl_4_∙3H_2_O and PEI solution turned red, indicating the formation of gold nanoparticles. The resultant AuPEINPs were purified by centrifugal filtration using 50 kDa MW cutoff membrane filters (repeated twice) to remove excess PEI. Synthesized AuPEINPs were characterized by UV-VIS spectroscopy using an Optizen POP (Mecasys, South Korea) instrument and by transmission electron microscopy (TEM) using a JEM-1200EX microscope at an accelerating voltage of 300 kV. The amount of N and C bound to the gold nanoparticle surface was calculated by elemental analysis (FlashEA 1112 NC analyzer, Thermo Fisher).

### Preparation of covalently bound polyethylenimine-gold nanoparticles (AuMUAPEINPs)

11-mercaptoundecanoic acid-polyethylenimine conjugates (MUAPEI) were prepared by amide bond formation. Briefly, 1 mmol of 11-mercaptoundecanoic acid and 1 mmol of N-hydroxysuccinimide were dissolved in 10 mL of dimethylformamide at room temperature, and 1 mmol of dicyclohexylcarbodiimide dissolved in 10 mL of dimethylformamide was added with stirring. Dicyclohexylurea precipitated from the reaction mixture after a few hours and the reaction mixture was allowed to stir for a total of 12 h. After 12 h, the reaction mixture was diluted with ethyl acetate, and the precipitated dicyclohexylurea was removed by filtration. After washing with brine, the ethyl-acetate layer was dried over anhydrous Na_2_SO_4_. Ethyl acetate was removed by evaporation and the obtained solid was dissolved in chloroform and again filtered to remove residual dicyclohexylurea. The filtrate was then washed three times with saturated potassium carbonate and brine and dried over Na_2_SO_4_. After evaporation of the solvent, 1-[(11-sulfanylundecanoyl)oxy] pyrrolidine-2,5-dione was obtained, and its structure was confirmed by mass spectral analysis.

Then 0.04 mmol of PEI was dissolved in 10 mL of tetrahydrofuran and added dropwise to 0.2 mmol of 1-[(11-sulfanylundecanoyl)oxy] pyrrolidine-2,5-dione dissolved in 10 mL of tetrahydrofuran with stirring. A white precipitate of MUAPEI formed instantaneously and the solution was stirred for 12 h. A solid product was obtained after centrifugation, and washed with tetrahydrofuran to remove excess reactants. After evaporation of the solvent, the solid product was resuspended in 25 mL of water, and filtered through 0.2 μm membrane filters. In the filtrate, 1.4 mM HAuCl_4_∙3H_2_O was dissolved and stirred vigorously for 30 min at room temperature. After 30 min, 1 mL of 10 mM NaBH_4_ solution was added and stirred overnight. Upon addition of NaBH_4_ solution, the yellow color of the mixed solution turned pink, indicating the formation of gold nanoparticles. The resultant AuMUAPEINPs were purified by centrifugal filtration using 50 kDa MW cutoff membrane filters (repeated twice) to remove excess MUAPEI. The synthesized AuMUAPEINPs were characterized by UV-VIS spectroscopy using an Optizen POP (Mecasys, South Korea) instrument and by transmission electron microscopy (TEM) using a JEM-1200EX microscope at an accelerating voltage of 300 kV. The amount of S, N, and C bound to the gold nanoparticle surface was calculated by elemental analysis (FlashEA 1112 NC analyzer, Thermo Fisher).

### Transmission electron microscopy (TEM) analysis for primary size determination

The primary sizes of AuPEINPs and AuMUAPEINPs were measured by transmission electron microscopy (TEM) using a JEM-1200EX microscope at an accelerating voltage of 300 kV.

### Thermogravimetric analysis (TGA)

The amount of PEI and MUAPEI on the Au-based NPs surfaces was evaluated by thermogravimetric analysis (TGA) with a heating rate of 10 ^o^C min^−1^ in a flowing nitrogen atmosphere using a Thermogravimetric Analyser (SDT Q600 system). Thermograms were recorded the temperature range 27−800 °C. The grafting density of PEI and MUAPEI was estimated by using the formula,

No. of molecules per nm^2^ = (*f* × *Na* × *d* × ρ) /((*1* − *f*) × *M* × 6), where ρ is density of the spherical gold nanoparticles (19.32 g cm^−3^), *f* is the weight fraction of the organic ligands determined by TGA, *Na* is the Avogadro constant, *d* is the diameter of nanoparticle core and *M* is the molecular weight of the polymeric ligands as described by Ye *et al*.^86^.

### Preparation of nanoparticle-pDNA complexes

AuPEINP and AuMUAPEINP-pDNA complexes were prepared with different ratios of nanoparticle numbers to 1 μg of pDNA. Both nanovector and pDNA solutions were prepared in 50 μL deionized (DI) H_2_O and then combined to give a final volume of 100 μL. The polyplex solutions were then vortexed gently for 10 s and incubated for 30 min at room temperature.

### UV spectroscopy, dynamic light scattering, and zeta potential measurements

The UV-visible spectra of AuPEINPs, AuMUAPEINPs, and nanoparticle-pDNA complexes were acquired using an Optizen POP (Mecasys, South Korea) instrument. The hydrodynamic size and zeta potential of AuPEINPs, AuMUAPEINPs and nanoparticle-pDNA complexes were measured using a Zetasizer Nano ZS90 (Malvern Instruments, Ltd., UK) instrument. For AuPEINP-pDNA and AuMUAPEINP-pDNA complexes, 1.33 × 10^11^ and 0.53 × 10^11^ nanoparticles were used respectively for 1 μg of pDNA.

### Agarose gel retardation assay

The pDNA binding ability of the nanovectors was determined using agarose gel (1.0% w/v) retardation assays. Agarose gels were prepared in Tris-acetate-EDTA buffer containing ethidium bromide (0.1 mg/mL). CeO_2_/DODAB-pDNA complexes were prepared with increasing number of nanoparticles using 1 μg of pDNA. Gel electrophoresis was performed at 80 V and the migration of pDNA in the gel was analyzed using a UV transilluminator (UVP, Bio Doc-It).

### Cell culture

Human Wharton’s jelly derived mesenchymal stem cells (hMSCs) were obtained from the Bangkok stem cell company. MSCs were cultured with α-modification minimum essential medium (α-MEM) supplemented with 10% fetal bovine serum (FBS) and 100 U/mL penicillin-streptomycin in a humidified atmosphere (5% CO_2_, 37 °C). Cell culture medium was exchanged every three days. All experiments were performed with cells after the fifth passage.

### Gene transfection and luciferase reporter assay

hMSC cells were seeded (5 × 10^4^ cells/well) into 24-well, flat bottom culture plates and incubated overnight at 37 °C in a 5% CO_2_ incubator. When hMSC cells reached 60–70% confluence, transfections were performed by adding 1 μg of pGL3 to each well. Prior to transfection, the medium was exchanged with 400 μL of fresh α-MEM without FBS and antibiotics. Subsequently, 100 μL of solutions containing nanoparticle-pGL3 complexes (AuPEINP-pGL3 or AuMUAPEINP-pGL3), Lipofectamine-pGL3, or PEI-pGL3 complexes were added to cells and incubated for 6 h in a humidified incubator at 37 °C in the presence of 5% CO_2_. The medium was then replaced with fresh medium containing 10% FBS and antibiotics and cultured for another 48 h. Luciferase activity was measured using a Luciferase assay following to the manufacturer’s (Promega) protocol. The efficiency of gene delivery efficiency is expressed in relative light units per gram of total protein (RLU/g).

### Transfection efficiency of nanoparticles complexed with expression plasmids encoding C/EBPβ

Cells were seeded and transfected as described above, with the exception that pEGFP-C/EBPβ was used instead of pGL3. After 48 h, cells were imaged using an inverted fluorescence microscope. The efficiency of gene delivery was quantified by flow cytometry using FACS Calibur and FACS data were analyzed with Cell Quest software. Gene transfection efficiency was also determined by western blot analysis of EGFP and C/EBPβ.

### Western blot analysis

Cells were lysed in radioimmunoprecipitation assay (RIPA) lysis buffer containing protease and phosphatase inhibitors. Equal amounts of protein were resolved by 10% sodium dodecyl sulfate-polyacrylamide gel electrophoresis (SDS-PAGE) and electrophoretically transferred to PVDF membranes. Membranes were blocked at room temperature with 5% non-fat dry milk for 2 h to prevent non-specific binding, and then incubated with specific primary antibodies overnight at 4 °C. Immunoreactivity was detected through sequential incubation with horseradish peroxidase-conjugated secondary antibodies and enhanced chemiluminescence reagents.

### Adipocyte differentiation

hMSCs were transfected with AuPEINP-pEGFP-C/EBPβ, AuMUAPEINP-pEGFP-C/EBPβ, or Lipofectamine-pEGFP-C/EBPβ for 6 h and then incubated with fresh α-MEM medium containing 10% FBS and antibiotics for 12 h. Cells were then cultured in adipogenic differentiation medium (ADM) supplemented with α-MEM, 10% FBS, 100 μM indomethacin (Sigma–Aldrich), 10 μg/mL insulin (Sigma–Aldrich), 0.5 mM isobutylmethylxanthine (Sigma–Aldrich), and 1 μM dexamethasone (Sigma–Aldrich). Non-transfected (control) cultures were maintained either in growth media or in ADM. Cultured cells were maintained for either 7 or 14 days in a humidified incubator at 37 °C and 5% CO_2._ Media was exchanged every 3 days.

### Staining with Oil Red O

Oil Red O stock solution was prepared by dissolving 0.7 g of Oil Red O (MP Biomedicals, OH, USA) in 200 mL of isopropanol, stirred overnight, then filtered through 0.2 μm filters and stored at 4 °C. Oil Red O working solution was prepared by mixing 6 parts of Oil Red O stock solution with 4 parts of water (by volume), which was cooled to room temperature and filtered through 0.2 μm filters. For staining, cells were washed with PBS, fixed with 4% paraformaldehyde, then washed with H_2_O and incubated with 60% isopropanol for 5 min. After 5 min, isopropanol was removed and the wells were dried completely. Oil Red O working solution was added and incubated for 10 min, which was then washed three times with H_2_O. Stained cells were imaged using an inverted microscope. For quantitative analysis, after staining the cells, Oil Red O was eluted with 100% isopropanol and the absorbance was measured at 500 nm.

### Gene expression analysis

Total mRNA from treated cells was extracted using an RNeasy Mini Kit (Qiagen) and cDNA was synthesized using a QuantiTect Reverse Transcription Kit (Qiagen) in a final volume of 20 μL, following the manufacturer’s instructions. All gene transcripts were measured in triplicate by qRT-PCR on a LightCycler apparatus, using LightCycler^®^ FastStart DNA Master SYBR Green I with an AB Applied Biosystems instrument. The primer sequences used to amplify each gene are shown in Supplementary Table 4. The relative quantification of gene expression was analyzed by the 2-ddCt method. In all experiments, Gapdh mRNA was used as an internal standard.

### Transmission electron microscopy (TEM) analysis for cellular localization of nanoparticle-DNA complexes

To check the cellular internalization of the nanoparticles-pDNA complexes, we tracked the intracellular distribution of the nano-complexes within the cells by TEM analysis following transfection of the cells with AuPEINPs-pDNA and AuMUAPEINPs-pDNA for 12 hrs. After treatment, hMSCs were washed, fixed, again washed, and then dehydrated in EtOH (70% > 80% > 90% > 95% > 100%). After that, cells were embedded in Epon-Araldite mix solution and blocked at 60 °C in a vacuum drying oven (Yamoto, DPF-31) for 36 h. First, semi-thin slides were made using an ultramicrotome (LKB-2088) and stained with 1% toluidine blue (1% borax) on a 60 °C hot plate for 2 min. After that, to observe the cell micro-structures, we made ultra-thin slices and stained with uranyl acetate and lead citrate. Examination of sections was performed with a transmission electron microscope operated at 100 kV.

### Sample Preparation for inductively coupled plasma mass spectrometry (ICP-MS) measurements

For the quantitative determination of Au content in the cellular uptake study, hMSCs were treated with AuPEINPs (1.33 × 10^11^) and AuMUAPEINPs (0.53 × 10^11^) complexed with 1 μg pDNA for 6 hrs. After that, cells were extensively washed to remove free and weakly bound nanoparticles-pDNA complexes. Then the cell pellet was dried and weighed. The cell pellet was then digested in 8 mL of aqua regia at 180 °C temperature for 4 hours. After that 20 mL of distilled water was added and allowed to vaporize the acid at 185 °C temperature until the volume of the solution reduced to 2~3 mL. After cooling the solution at room temperature, the volume of the sample solution wasmade upto 15 mL. The cellular uptake of Au in the sample solutions were measured on a Perkin-Elmer SCIEX, NexION 350D ICP-mass spectrometer. We have treated approximately 5.4 × 10^5 ^cells with the nanoparticle-pDNA complexes and the amount of gold per cell was converted in to the number of gold nanoparticles per cell by using the formula;


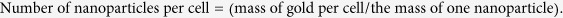


Similarly, the uptake efficiency was calculated by using the formula;


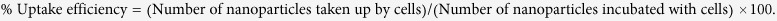


### Measurement of cell membrane integrity

Cell membrane integrity was assessed by lactate dehydrogenase (LDH) release into the media using an LDH Assay Detection kit (Takara Bio Inc., Tokyo, Japan), according to the protocol of the manufacture, by measuring the absorbance at 490 nm using a microplate reader.

### Intracellular distribution of nanoparticle-Cy3-labeled DNA complexes

To determine the intracellular distribution of nanovector-DNA complexes, cells were incubated with nanoparticle-Cy3-labeled DNA complexes (1.33 × 10^11^ AuPEINPs and 0.53 × 10^11^ AuMUAPEINPs were used for 1 μg of Cy3-DNA) for 12 h. Treated cells were then incubated with LysoTracker Green for 30 min and washed three times with PBS to eliminate background signals. Stained cells were imaged using a confocal laser scanning microscope.

### Cellular uptake pathways

Cells were seeded into 24-well culture plates as described previously. When cells reached 60–70% confluence, they were pretreated with several endocytosis inhibitors, such as with 1 mM methyl-β-cyclodextrin or 5 μg/mL chlorpromazine, or with a macropinocytosis inhibitor—65 μM LY294002 (Sigma–Aldrich, St. Louis, MO, USA) for 60 min. Cells were then transfected with nanoparticle-pGL3 complexes for 6 h, as described previously. Luciferase activity was measured after 48 h using a Luciferase assay following the manufacturer’s (Promega) protocol.

### Cytotoxicity assay

Cells were seeded (1.5 × 10^4 ^cells/well) into 96-well, flat bottom culture plates and incubated for 24 h at 37 °C in a 5% CO_2_ incubator. Media was replaced with fresh α-MEM containing no FBS and antibiotics. Cells were then treated with nanoparticle-pEGFP-C/EBPβ Lipofectamine 2000-pEGFP-C/EBPβ and PEI-pEGFP-C/EBPβ complexes for 6 h in a humidified incubator at 37 °C in the presence of 5% CO_2_. After 6 h, the medium was replaced with fresh medium containing 10% FBS and antibiotics. Cell viability assays were performed using the Cell Counting Kit-8 (CCK-8, Dojindo Laboratories, Kumamoto, Japan) after 48, 72 and 96 h. Absorbance was measured at a wavelength of 450 nm using a microtiter plate reader (Multiskan FC, Thermo Fisher Scientific Inc., Waltham, MA, USA).

### Statistical analysis

All experiments were performed at least in triplicate, and statistical analyses were performed using one-way analysis of variance (ANOVA) followed by a Student’s *t*-test. The level of significance was set at *p < 0.05, **p < 0.01, and ***p < 0.001.

## Additional Information

**How to cite this article**: Joydeep, D. *et al*. Efficient delivery of C/EBP beta gene into human mesenchymal stem cells via polyethylenimine-coated gold nanoparticles enhances adipogenic differentiation. *Sci. Rep.* **6**, 33784; doi: 10.1038/srep33784 (2016).

## Supplementary Material

Supplementary Information
