# Corrigendum: Efficient delivery of C/EBP beta gene into human mesenchymal stem cells via polyethylenimine-coated gold nanoparticles enhances adipogenic differentiation

**DOI:** 10.1038/srep37480

**Published:** 2016-11-21

**Authors:** Joydeep Das, Yun-Jung Choi, Hideyo Yasuda, Jae Woong Han, Chankyu Park, Hyuk Song, Hojae Bae, Jin-Hoi Kim

Scientific Reports 6: Article number: 33784; 10.1038/srep33784 published online: 09 28 2016; updated: 11 21 2016.

The original version of this Article contained errors in the spelling of the author Joydeep Das, which was incorrectly given as Das Joydeep.

These errors have now been corrected in the PDF and HTML versions of the Article.

