# Supplementary material for: Efficient delivery of C/EBP beta gene into human mesenchymal stem cells via polyethylenimine-coated gold nanoparticles enhances adipogenic differentiation
Source: Sci Rep. 2016 Sep 28;6:33784. doi: 10.1038/srep33784 (PMC5039411; doi:10.1038/srep33784)
Supplement: Supplementary Information [file srep33784-s1.doc]

**Efficient delivery of C/EBP beta gene into human mesenchymal stem cells via polyethylenimine-coated gold nanoparticles enhances adipogenic differentiation**

Joydeep Das^1^, Yun-Jung Choi^1^, Hideyo Yasuda^1^, Jae Woong Han^1^, Chankyu Park^1^, Hyuk Song^1^, Hojae Bae^2^, and Jin-Hoi Kim^1^*

^1^Dept. of Stem Cell and Regenerative Biology, Humanized Pig Research Center (SRC), Konkuk University, Seoul 143-701, South Korea

^2^Dept. of Bioindustrial Technologies, College of Animal Bioscience and Technology, Konkuk University, Seoul 143-701, South Korea

**Running title:** Gold nanoparticle mediated gene delivery

**Corresponding author:**

**Jin-Hoi Kim*:** [jhkim541@konkuk.ac.kr](mailto:jhkim541@konkuk.ac.kr); Fax: +82-2-458-5414

Department of Animal Biotechnology,

Humanized Pig Research Center (SRC),

Konkuk University, Seoul 143-701, South Korea

**Supplementary Informations:**

**Supplementary Table 1: Elemental analysis of AuPEINPs and AuMUAPEINPs**

| **Nanovector** | **% Nitrogen** | **% Carbon** | **% Sulphur** |
| --- | --- | --- | --- |
| **AuPEI** | **9.435** | **22.548** | **0** |
| **AuMUAPEI** | **8.132** | **24.106** | **0.216** |

**Supplementary Table 2: TGA weight loss and grafting density**

| **Nanovector** | **% Weight loss of**  **organic ligand** | **No. of molecules per nanoparticle** | **Grafting density**  **(No. of molecules nm^-2^)** |
| --- | --- | --- | --- |
| **AuPEI** | **52.50** | **34** | **0.43** |
| **AuMUAPEI** | **70.19** | **412** | **1.62** |

**Supplementary Table 3: Hydrodynamic size and zeta potential of AuPEI-pDNA and AuMUAPEI- pDNA complexes in water. All values are expressed as mean ± SD.**

| **Vectors/Complexes** | **Diameter (nm)** | **Zeta potential (mV)** |
| --- | --- | --- |
| **AuPEINPs (1.33x 10^11^) -pDNA (1 μg)** | **71.23± 6.13** | **28.6± 2.7** |
| **AuMUAPEINPs (0.53x 10^11^) -pDNA (1 μg)** | **89.31± 0.91** | **21.70± 0.60** |

**Supplementary Table 4: Primer sequences used for PCR**

| **Primer name** | **Primer sequence** | **Annealing** |
| --- | --- | --- |
| **C/EBPβ** | **F: cacagcgacgactgcaagatcc**  **R: cttgaacaagttccgcagggtg** | **61°C** |
| **PPARγ2** | **F: Tgtctcataatgccatcaggtttg**  **R: gataacgaatggtgatttgtctgtt** | **58°C** |
| **AP2** | **F: accaggaaagtggctggcat.**  **R: caggtcaacgtcccttggct** | **58°C** |
| **GAPDH** | **F: cttttaactctggtaaagtgg**  **R: ttttggctcccccctgcaaat** | **58°C** |

**Supplementary Figure legends**

**Figure 1:** Gene map.

**Figure 2:** Mass spectra of 1-[(11-sulfanylundecanoyl)oxy] pyrrolidine-2,5-dione.

**Figure 3:** UV-VIS spectra of AuPEINPs (a) and AuMUAPEINPs (b) and their complexes with pDNA.

**Figure 4:** (a) Hydrodynamic diameter and (b) Zeta potential of AuPEINPs and AuMUAPEINPs. All values are expressed as mean ± SD.

**Figure 5:** Thermogravimetric analysis. First derivative of the weight loss as a function of temperature for bare MUAPEI.

**Figure 6: Oil Red O staining 14 days after transfecting the C/EBPβ gene** (a) Oil Red O staining of non-treated control cells cultured in growth media. (b) Oil Red O staining of non-treated control cells cultured in ADM. (c) Oil Red O staining of cells expressing the C/EBPβ gene transfected with Lipofectamine 2000 and cultured in ADM. (d) and (e) Oil Red O staining of cells expressing the C/EBPβ gene transfected with AuPEINPs (1.06 × 10^11^ and 1.33 × 10^11^ number of particles, respectively) and cultured in ADM. (f) and (g) Oil Red O staining of cells expressing the C/EBPβ gene transfected with AuMUAPEINPs (0.35 × 10^11^ and 0.53 × 10^11^ number of particles, respectively) and cultured in ADM. (h) Quantitative analysis of lipid droplet formation. All values are expressed as mean ± SD. *p < 0.05, **p < 0.01, and ***p < 0.001 versus the control non-transfected group. #p < 0.05, ##p < 0.01, and ###p < 0.001 versus Lipofectamine transfected group.

**Figure 7:** Optical microscopic images of hMSCs after incubation with the AuPEINP (0.8 x 10^11^- 2.65 x 10^11^)-pDNA (1 μg) complexes in the medium for 6 h.

**Figure 8:** Optical microscopic images of hMSCs after incubation with the AuMUAPEINP (0.35 x 10^11^- 1.05 x 10^11^)-pDNA (1 μg) complexes in the medium for 6 h. Arrows indicate nanoparticle-pDNA complex aggregation.

**Figure 9:** LDH release in hMSCs, after treatment with different concentrations of AuPEINPs and AuMUAPEINPs for 12 h. All values are expressed as mean ± SD.


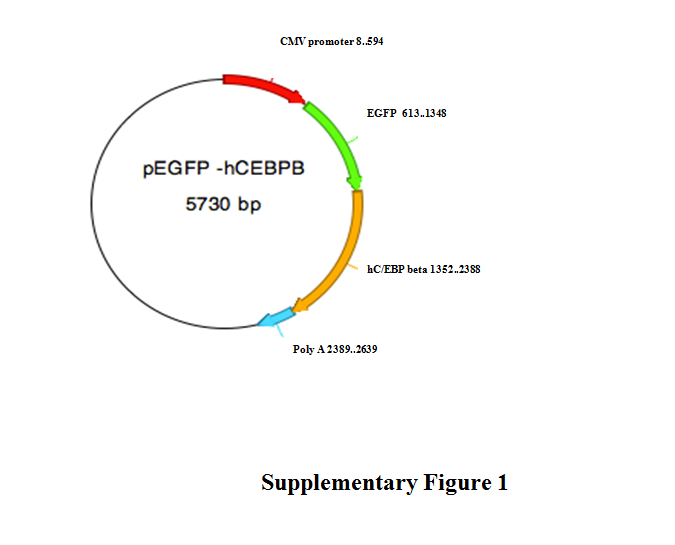


**
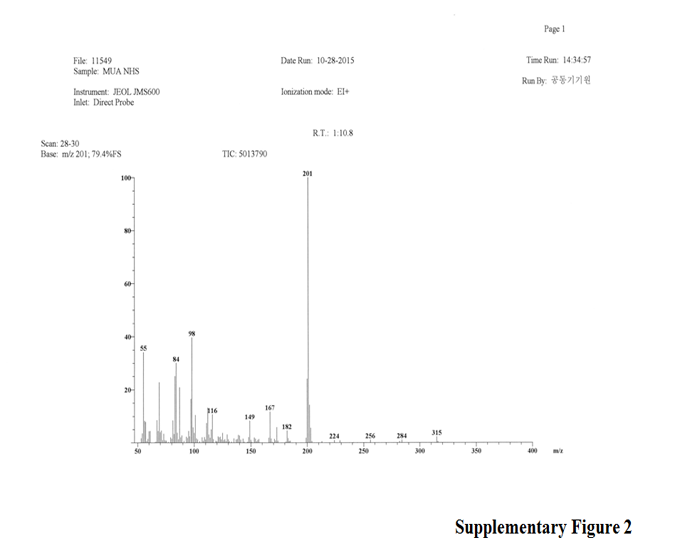
**

**
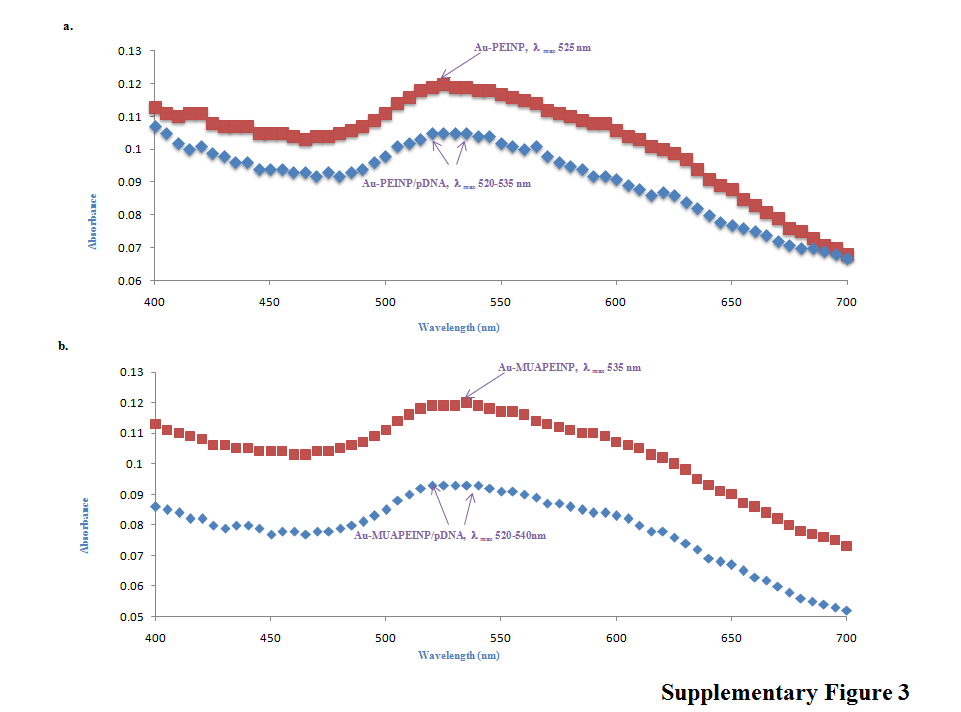
**

**
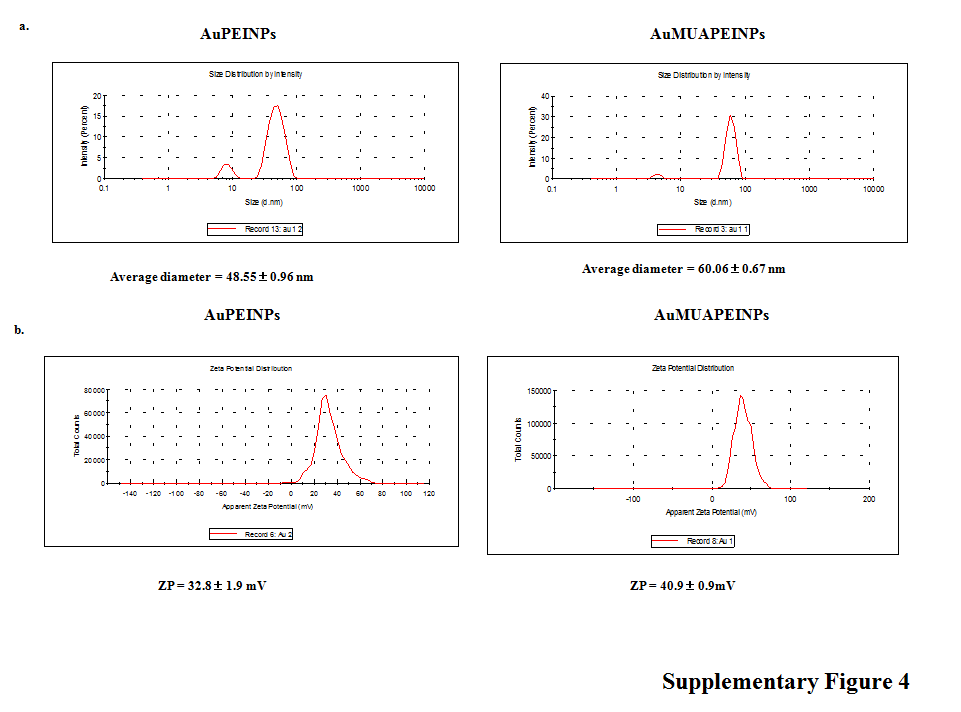
**

**
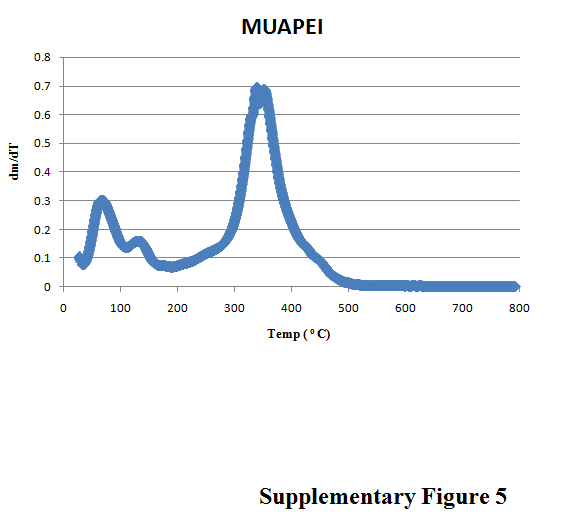
**

**
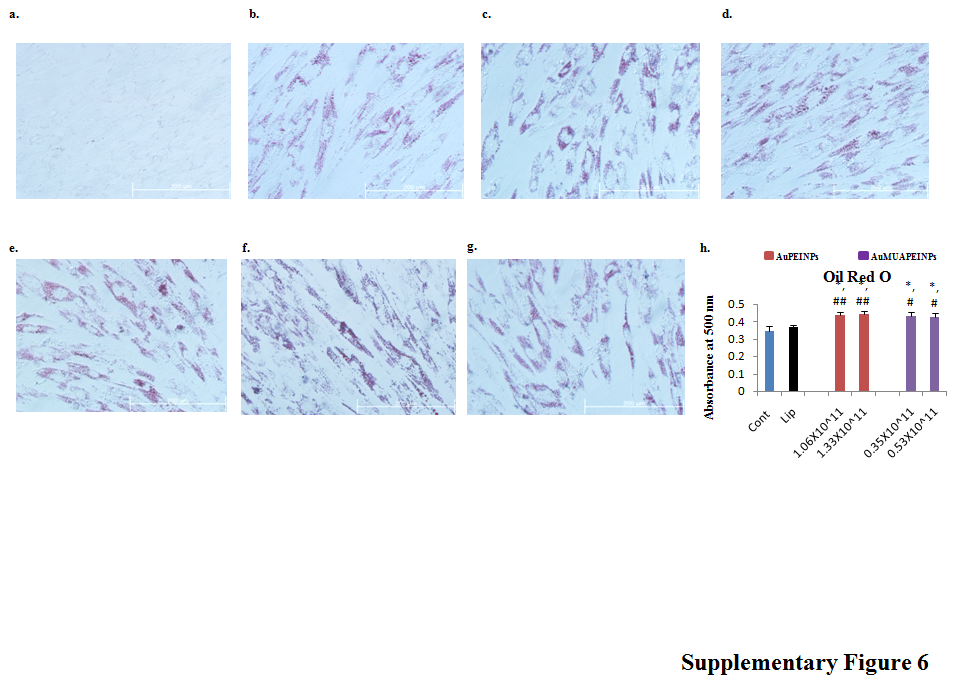
**

**
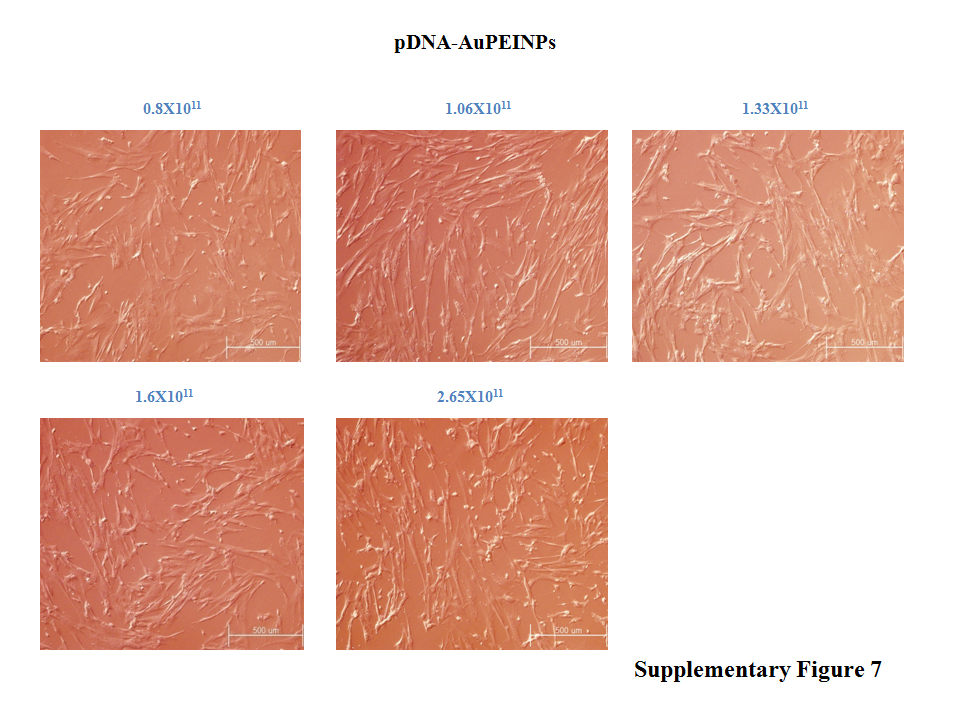
**

**
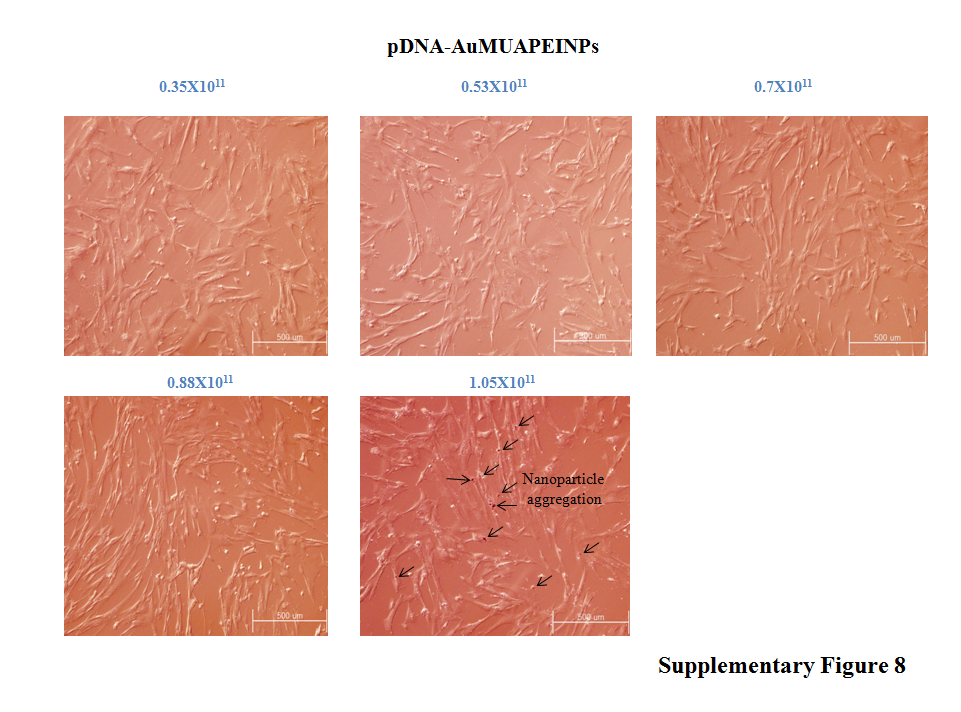
**

**
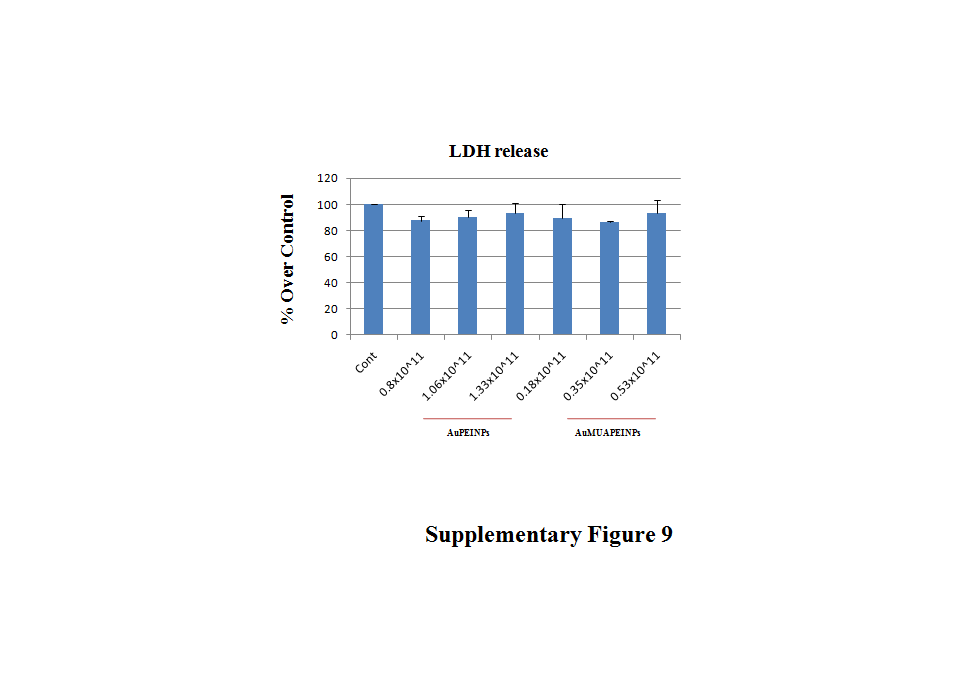
**
